# Sequential Application of Antioxidants Rectifies Ion Imbalance and Strengthens Antioxidant Systems in Salt-Stressed Cucumber

**DOI:** 10.3390/plants9121783

**Published:** 2020-12-16

**Authors:** Mahmoud F. Seleiman, Wael M. Semida, Mostafa M. Rady, Gamal F. Mohamed, Khaulood A. Hemida, Bushra Ahmed Alhammad, Mohamed M. Hassan, Ashwag Shami

**Affiliations:** 1 Plant Production Department, College of Food and Agriculture Sciences, King Saud University, P.O. Box 2460, Riyadh 11451, Saudi Arabia; 2 Department of Crop Sciences, Faculty of Agriculture, Menoufia University, Shibin El-Kom 32514, Egypt; 3 Horticulture Department, Faculty of Agriculture, Fayoum University, Fayoum 63514, Egypt; wms00@fayoum.edu.eg; 4 Botany Department, Faculty of Agriculture, Fayoum University, Fayoum 63514, Egypt; mmr02@fayoum.edu.eg (M.M.R.); gfm00@fayoum.edu.eg (G.F.M.); 5 Botany Department, Faculty of Science, Fayoum University, Fayoum 63514, Egypt; kah00@fayoum.edu.eg; 6 Biology Department, College of Science and Humanity Studies, Prince Sattam Bin Abdulaziz University, Al Kharj Box 292, Riyadh 11942, Saudi Arabia; b.alhamad@psau.edu.sa; 7 Department of Biology, College of Science, Taif University, P.O. Box 11099, Taif 21944, Saudi Arabia; m.khyate@tu.edu.sa; 8 Department of Genetics, Faculty of Agriculture, Menoufia University, Sheben El-Kom, Menoufia 32514, Egypt; 9 Biology Department, College of Sciences, Princess Nourah Bint Abdulrahman University, Riyadh 11617, Saudi Arabia; AYShami@pnu.edu.sa

**Keywords:** sequential application of antioxidants, salinity, *Cucumis sativus*, photosynthetic efficiency, antioxidant defense systems

## Abstract

Exogenous antioxidant applications enable salt-stressed plants to successfully cope with different environmental stresses. The objectives of this investigation were to study the effects of sequential treatments of proline (Pro), ascorbic acid (AsA), and/or glutathione (GSH) on 100 mM NaCl-stressed cucumber transplant’s physio-biochemical and growth traits as well as systems of antioxidant defense. Under salinity stress, different treatment of AsA, Pro, or/and GSH improved growth characteristics, stomatal conductance (gs), enhanced the activities of glutathione reductase (GR), superoxide dismutase (SOD), ascorbate peroxidase (APX), and catalase (CAT) as well as increased contents of AsA, Pro, and GSH. However, sequential application of antioxidants (GSH-Pro- AsA) significantly exceeded all individual applications, reducing leaf and root Cd^2+^ and Na^+^ contents in comparison to the control. In plants grown under NaCl-salt stress, growth characteristics, photosynthetic efficiency, membrane stability index (MSI), relative water content (RWC), contents of root and leaf K^+^ and Ca^2+^, and ratios of K^+^/Na^+^ and Ca^2+^/Na^+^ were notably reduced, while leaf contents of non-enzymatic and enzymatic antioxidants, as well as root and leaf Cd^2+^ and Na^+^ concentrations were remarkably increased. However, AsA, Pro, or/and GSH treatments significantly improved all investigated growth characteristics, photosynthetic efficiency, RWC and MSI, as well as AsA, Pro, and GSH, and enzymatic activity, leaf and root K^+^ and Ca^2+^ contents and their ratios to Na^+^, while significantly reduced leaf and root Cd^2+^ and Na^+^ contents.

## 1. Introduction

Cucumber (*Cucumis sativus* L.) is one of the most economically important and widely distributed vegetable crops, as well as being useful for human health and nutrition because it has several important mineral nutrients and vitamins and has reasonable amounts of proteins and carbohydrates [1]. In addition, it has many natural antioxidants and vitamins used as a nutritive source and for medical purposes such as headaches and anti-acne lotions due to its analgesic activities [2]. Also, cucumber can have antioxidants phenolic, especially flavonoids can be involved in reactive oxygen species (ROS) detoxification.

Cucumber has been found to be moderately sensitive to salinity [3]. In particular, salt stress causes inhibition of cucumber plant growth and productivity through affecting the key physiological and biochemical processes [4,5,6,7]. Due to the climatic changes occurring presently, salinization of plant-growing media is gradually aggravated, which will lead to further inhibition and loss in plant growth and yield, respectively. Therefore, considerable attention has been paid to boost salinity tolerance in cucumber plants by using many exogenous supports [6,8,9].

Generally, salinity negatively affects growth and restricts the productivity and quality of crops, especially in arid and semi-arid agricultural regions [10,11,12,13,14,15]. Plant growth and productivity are declined at varying degrees subjecting to the salt stress levels [1,16,17]. Salt stress causes plant performance reduction through the dysfunction occurred in the biochemical and physiological and processes, and the antioxidant defenses as a result of ROS excessive production such as ^1^O_2_, O_2_^•−^, H_2_O_2_, and OH^‒^, as well as the instabilities of cell membranes and lipid peroxidation occurred due to stress of increased Na+ ions along with increased ROS [18,19,20,21,22].

Plants have different antioxidative mechanisms for ROS scavenging through inspiring the antioxidative enzymes for instance CAT, GR, SOD, and APX, which are functioned along with the non-enzymatic antioxidants to mitigate the adverse impacts of different stresses in plant species [23,24,25]. The main non-enzymatic plant antioxidants are ascorbate, glutathione, carotenoids, tocopherols, and phenolic compounds. Non-enzymatic antioxidants such as AsA, GSH, and Pro can represent a fundamental portion of the endogenous defense mechanisms and adaptive strategies to fight the stress. They are often insufficient to cope with stress; therefore, plants are exogenously provided by these antioxidants singly or in combinations to promote and enhance their antioxidant defense systems in contrast to different environmental stresses, including salinity [26,27,28]. As reported in these works, the exogenous treatments (i.e., AsA, Pro, and GSH) applied singly or in sequences had boosted plant performance (i.e., growth and yield) through a positive stimulation of the key physio-biochemical and molecular attributes and increasing the capacity of antioxidant defenses. In addition, the above works have reported ROS detoxification, cell membrane stability, ion balance, and Na^+^ ions decline.

To our knowledge, only little previous works [24,28,29] showed that the sequential application of Pro, AsA, and GSH is proved to be more effective strategy for plants to efficiently cope with various abiotic stresses (i.e., heavy metals, drought, and salinity) than their individual or combined applications. Therefore, the aims of the current investigation were to assess the effects of exogenously applied AsA, Pro and GSH in a sequence method in comparison with individual treatments on the growth, efficiency of photosynthesis, tissue water content, and membrane health in terms of MSI and the contents of Cd^2+^ and Na^+^ as well as non-enzymatic and enzymatic defenses of cucumber transplants.

## 2. Materials and Methods

### 2.1. Experimental Setup

Seeds of cucumber (hybrid Bahi^®^) were separated to five groups, each consisted of 80 seeds. Then, seeds were soaked in distilled water (group 1), 0.5 mM AsA solution (group 2), 0.5 mM Pro solution (group 3), or 0.5 mM GSH solution (group 4). The duration of soaking was 4 h. Furthermore, seeds of group number 5 were soaked in sequential application of different antioxidants as follows: 0.5 mM AsA for 90 min, 0.5 mM Pro for 80 min, and then 0.5 mM GSH for 70 min in a sequence, respectively. Concentrations of antioxidants applied singly or in AsA-Pro-GSH and soaking duration were selected according to our data (not shown) of a preliminary study. In addition, AsA-Pro-GSH was generated greatest response among different tested sequences of our preliminary study (data not shown).

In each group, the seeds were divided into two sub-groups (i.e., n = 40 seeds) to represent 10 treatments. After germinating the seeds of all groups, irrigation was applied using a distilled water for all first sub-groups in the same time, while all second sub-groups were irrigated with 100 mM NaCl solution. Foam trays (209 cells) were used for the current work conducted on 28 May 2017 for 28 d. Trays were arranged in an open greenhouse under which the conditions were 62–68% humidity, 29 ± 3/19 ± 1 °C day/night temperatures, and average 13/11 h day/night length. The light intensity was the intensity of natural sunlight throughout the season (28th of May–24th of June). Peat moss and vermiculite mixture (1:2 *v/v*) was a germinating and growing medium of transplants. The completely randomized designs was used for the experiment with three foam trays/replicates for each treatment. Twenty-eight days after sowing, transplants of cucumber were collected for different measurements of morphology and physiological traits and biochemical attributes.

### 2.2. Measurements

After 28 days, seedlings (n = 20) were randomly selected from each treatment to record growth attributes i.e., leaf area, shoot length, stem diameter, and shoot FW (fresh weight) and DW (dry weight). Leaf area was determined as detailed by [30].

Stomatal conductance (gs) mmol^−2^ S^−1^ was measured by leaf porometer (Decagon Devices Inc., Pullman, WA, USA). The measurements were conducted 10, 14, 18, and 22 days after onset of NaCl applications from 7:00 am: 5:00 pm with 2 h intervals. SPAD chlorophyll meter (SPAD-502; Minolta, Osaka, Japan) was used to measure relative contents of chlorophyll. Chlorophyll fluorescence (performance index, PI; = maximum quantum yield of PSII = efficiency of the photosystem 2, Fv/Fm; Fv/F0), as a convenient tool to assess photosynthetic efficiency, was determined according to [31] using Handy PEA (Hansatech Instruments Ltd., Kings Lynn, UK). Leaves relative water content (RWC) and cell membrane stability index (MSI) were measured according to [32].

The method detailed by [33] was followed to determine leaf content of free proline after extraction of 500 mg fresh leaf using 3%, *v*/*v*, sulphosalicylic acid. Following the [34] method, leaf AsA was extracted and its content was determined. Leaf GSH content was determined as detailed in the [35] method. Protein content and activity of SOD (EC 1.15.1.1) was analyzed as described by Bradford [36] and Kono [37] respectively. CAT (EC 1.11.1.6) activity was analyzed as described by Aebi [38]. Potassium phosphate (KH_2_PO_4_; a buffer) and hydrogen peroxide (H_2_O_2_; a substrate) were used, and the absorbance was read using spectrophotometer at 240 nm. The activity of APX (EC 1.11.1.11) was analyzed using the method of [39] and the absorbance was read using spectrophotometer at 290 nm. GR (EC 1.6.4.1) activity was analyzed and the NADPH oxidation was monitored for three absorbance readings recorded at 340 nm [39]. 

A weight of 100 mg dried powdered leaf or root samples was digested for 12 h by using 80% HClO4 (2 mL) + concentrated H2SO4 (10 mL). The digested samples were then diluted each to 100 mL. The digested leaf samples were used to analyze the concentrations of K^+^ and Ca^2+^ via flame photometry [40]. In addition, the digested leaf and root samples were used to analyze Cd^2+^ contents via Atomic Absorption Spectrophotometer (a Perkin-Elmer, Model 3300) as detailed in [41] method.

### 2.3. Statistical Analysis 

The data obtained from the effects of exogenously applied AsA, Pro and GSH in a sequence method in comparison with individual treatments on growth, physiological and biochemical traits as well as Cd^2+^ and Na^+^ contents of cucumber transplants gown under 100 mM NaCl-salt stress were statistically analyzed using the GLM procedure of Gen STAT (version 11; VSN International Ltd., Oxford, UK). Least significant differences (LSD) was calculated to compare the differences among means at 5% probability (*p* ≤ 0.05).

## 3. Results

**Figure 1:**
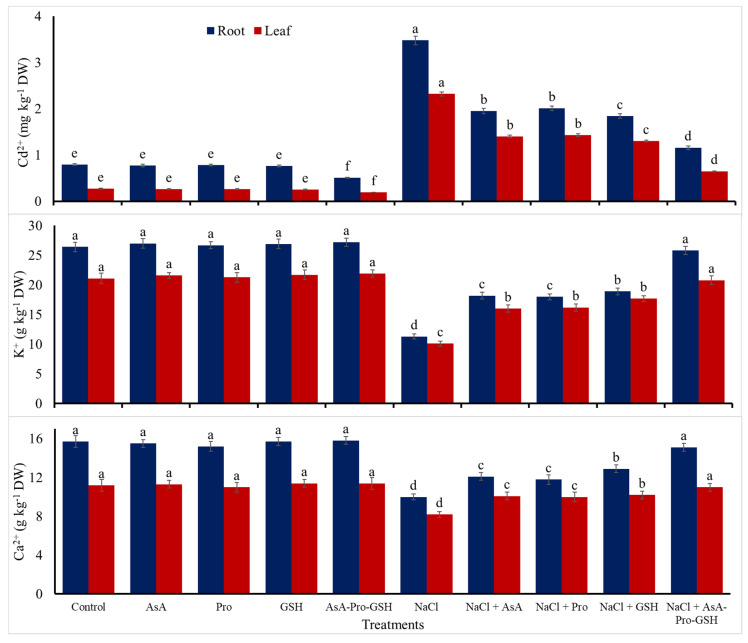
Effects of seed single or sequential application of antioxidants on Cd^2+^, K^+^ and Ca^2+^ contents in roots and leaves of 100 mM NaCl-salt-stressed cucumber transplants. Means with the same letter within each trait are not significantly differed at *p* ≤ 0.05. AsA = ascorbic acid, Pro = proline, GSH = glutathione, and AsA-Pro-GSH = sequential application of different antioxidants.

**Figure 2:**
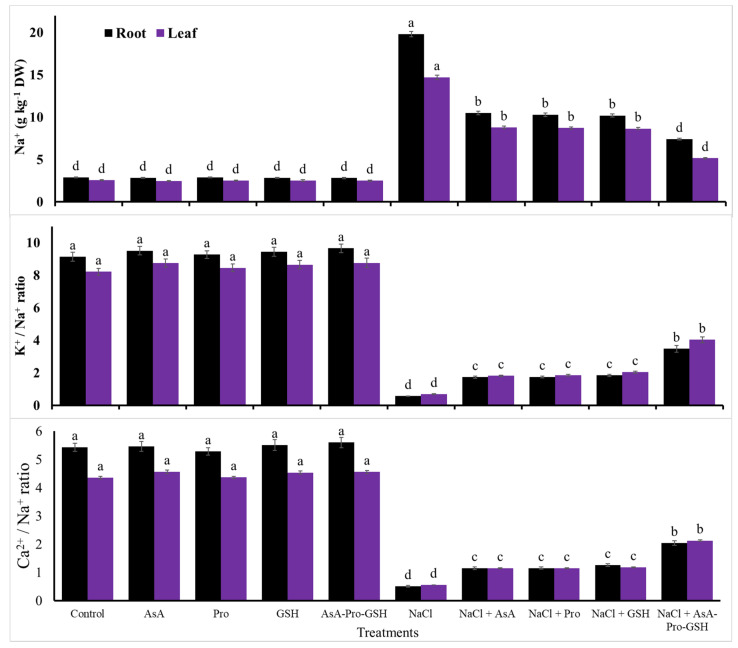
Effects of seed single or sequential application of antioxidants on the content of Na^+^ and its relation to K_+_ and Ca^2+^ in roots and leaves of 100 mM NaCl-salt-stressed cucumber transplants. Means with the same letter within each trait are not significantly differed at *p* ≤ 0.05. AsA = ascorbic acid, Pro = proline, GSH = glutathione, and AsA-Pro-GSH = sequential application of different antioxidants.

Concerning the antioxidant applications under normal conditions, the three antioxidants; AsA, Pro, and GSH that applied individually for cucumber seed improved all of the investigated growth characteristics (Table 1), gs (Table 2) and endogenous contents of AsA, Pro, and GSH (Table 3), as well as the enzymatic activities of SOD, CAT, GR, and APX (Table 4). On the other side, all of these individual antioxidant applications did not affect Fv/Fm, and PI and chlorophyll content (Table 2), RWC and MSI (Table 3), leaf and root contents of the poisonous elements Cd^2+^ and Na^+^, and the beneficial elements K^+^ and Ca^2+^ (Figure 1 and Figure 2). However, AsA-Pro-GSH treatment leads to better results for most of investigated parameters than those obtained with the individual applications, along with significant reductions of Cd^2+^ contents of leaves and roots upon comparing to the controls (Figure 1).

Regarding the stress impacts of salinity, treatment with 100 mM NaCl considerably suppressed cucumber transplant growth parameters compared to those grown under control. For instance, it reduced the length of plant shoot by 24.4%, leaf area by 26.3%, stem diameter by 16.7%, shoot FW by 17.5%, and shoot DW by 23.1% (Table 1), photosynthetic efficiency (i.e., gs by 26.4%, SPAD chlorophyll by 19.7%, Fv/Fm by 8.4%, and PI by 16.8%; Table 2), leaf RWC and MSI (by 15.0 and 16.6%, respectively; Table 3), respectively compared to the controls. However, treatment with 100 mM NaCl significantly increased leaf contents of antioxidants, which are non-enzymatic and have low-molecular-weights such as Pro by 22.2%, AsA by 95.3%, and GSH by 28.6% (Table 3), leaf enzymatic activities such as SOD by 8.7%, CAT by 26.1%, GR by 18.5%, and APX by 20.0%, respectively in comparison to the plants grown under control treatment (Table 4).

Treatment with 100 mM NaCl resulted in a reduction for leaf and root contents of Ca^2+^ and K^+^ by 26.8 and 36.3%, and 52.0 and 57.2% in comparison to controls, respectively (Figure 1). Moreover, it reduced the ratios of Ca^2+^/Na^+^ and K^+^/Na^+^ of leaves and roots by 87.2 and 90.6%, and 91.6 and 93.8% compared to the controls, respectively (Figure 2). Nevertheless, salt stress treatment with 100 mM NaCl resulted in an increments in the contents of root and leaf Cd^2+^ and Na^+^ by 340.1 and 759.3%, and 585.1 and 472.0%, respectively compared to the controls (Figure 1 and Figure 2).

However, the application of non-enzymatic antioxidants for cucumber seed attenuated the stress impacts of 100 mM NaCl salinity by modifying, positively, the above salt stress effects. Where compared to NaCl treatment, the individual use of each of AsA, Pro, and GSH for cucumber seed markedly elevated all of the investigated growth characteristics, photosynthetic efficiency, RWC, MSI, endogenous AsA, Pro, and GSH levels (further increase), and the activity of antioxidant enzymes; CAT, APX, GR, and SOD (further increase), the leaf and root Ca^2+^ and K^+^ contents and their ratios to Na^+^, while considerably reduced Cd^2+^ and Na^+^ contents in leaves and roots. However, AsA-Pro-GSH treatment significantly exceeded all of the individual applications for all of the investigated parameters. This AsA-Pro-GSH applied sequentially elevated the length of the shoot by 51.7%, total area of leaves by 49.8%, stem diameter by 23.3%, shoot FW by 41.8%, shoot DW by 60.0%, gs by 77.5%, SPAD chlorophyll by 22.5%, Fv/Fm by 10.5%, PI by 22.8%, RWC by 17.9%, and MSI by 20.8%. This best treatment also increased the activity of SOD by 52.0%, CAT by 41.4%, GR by 46.9%, and APX by 52.8%, the content of Pro by 163.6%, AsA by 162.3%, GSH by 227.8%, root K^+^ by 128.3%, leaf K^+^ by 105.9%, root Ca^2+^ by 51.0%, and leaf Ca^2+^ by 34.1%, and the ratio of root K^+^/Na^+^ by 510.5%, leaf K^+^/Na^+^ by 484.1%, root Ca^2+^/Na^+^ by 300.0%, and leaf Ca^2+^/Na^+^ by 280.4% compared to NaCl treatment without antioxidants. AsA, Pro, and GSH applied individually or sequentially were more noticeable under salt stress than their applications under the control condition, especially for the AsA-Pro-GSH applied sequentially.

## 4. Discussion

Salinity significantly limited cucumber transplant growth characteristics (Table 1). This growth limitation might be due to alterations in osmotic potential emerging from the limited availability of water [42]. An increased concentration of salts restricts the ability of the plant to absorb enough water and forces it to absorb Na^+^ and Cl^‒^ in excessive amounts, early emerging osmotic stress and accumulating ionic Na^+^ and Cl^‒^ stresses. This mainly impairs the metabolic processes, negatively affecting photosynthetic efficiency and limiting the growth [43]. However, sequence use of AsA-Pro-GSH conferred significant results, mitigating the devastating impacts of salinity stress. AsA, Pro, or GSH applied exogenously as individual treatments have been reported to overcome the disastrous impacts of salinity on metabolic processes related to plant growth [21,44,45,46]. However, the exogenous use of AsA, Pro, and GSH as AsA-Pro-GSH treatment has rarely been shown to attenuate the salt stress influences [28]. AsA, proline, and GSH as antioxidants have great power to counter and rectify the damages constructed by the salt-induced ROS. This enables stressed plants to qualify a complex antioxidative system to maximize defensive strategies in plant cells to tolerate the NaCl-induced oxidative stress [18,28,47]. Soaked seeds using AsA-Pro-GSH generated significantly better growth results than seeds soaked in individual AsA, Pro, or GSH solution. This may be attributed to that application of the three antioxidants in an integrative sequential method confers a balance for the so-called “AsA-GSH cycle” to effectively control the ROS radicals along with the osmoprotectant Pro to save water for cellular processes under salt stress in favor of plant growth. As proved for the AsA-Pro-GSH treatment in our earlier study [28], AsA was the most helpful when applied first because it has been involved in several biological activity types associated with oxidative stress resistance in plant cells, where it functions as a donor/acceptor in electron transport in the chloroplasts or at the cellular plasma membranes, in addition to acting as an antioxidant and enzymatic cofactor, etc. [48]. In chloroplasts, AsA is oxidized to monodehydroascorbate (MDA) in the presence of APX in the “Halliwell-Asada” pathway. MDA might trigger dehydroascorbate (DHA), which is reduced with MDA to cause regeneration of the ascorbate pool. This scavenging type can occur close to the PSII, leading to minimizing the ROS escape risk and/or their reacting with each other [49]. Also, the AsA acts as the “terminal antioxidant” due to the redox potential of the AsA/MDA pair (280 mv), which is lower than the redox potential of most bio-radicals [50]. These into the elucidation of [51] that the biosynthesis of AsA from hexose phosphate and its inclusion in protecting against the stress effects of photo-oxidation suggests that there could be links between the size of AsA pool and the photosynthesis process.

Under salt (100 mM NaCl) stress, photosynthesis efficiency attributes (i.e., gs, SPAD chlorophyll, Fv/Fm, and PI; Table 2) of cucumber transplants were significantly decreased. This may be due to the inhibited or insufficient nutrient uptake [52], which accompanied by the increased uptake of undesired elements (i.e., Na^+^ and Cd^2+^) as shown in our results (Table 4 and Table 5). It has also been explained that the diminished contents of chlorophyll could be attributed to the inhibited biosynthesis of chlorophyll and/or the increased chlorophyll-degrading enzyme chlorophyllase [53]. Since chlorophyll is reported as an indicator of oxidative damage, ROSs induced by salt also contribute to chlorophyll degradation and loss of pigment. Fv/Fm ratio reflects the master photochemistry of PSII capacity that is very susceptible to different stress impacts induced by environmental stressors [54]. The ratio of Fv/Fm shows, firmly, a noteworthy susceptibility to stress influences of salinity and it is reported as an indicator of photo-inhibition and/or other PS2 complexes injuries [55]. In the current work, our findings displayed that in the same time of which various antioxidant applications did not affect the photosynthetic efficiency attributes under normal conditions, they significantly improved these attributes in salt-stressed cucumber transplants, with a preference for the AsA-Pro-GSH treatment. The improvements in the values of SPAD chlorophyll, gs, PI, and Fv/Fm were positively reflected in transplants growth improvement. This may be elucidated according to the integrative roles giving integrative mode of actions of AsA, Pro, and GSH as a major mechanism in mitigating the impacts of salt stress in plants [28].

Our findings reported that RWC and MSI were markedly decreased under NaCl-salt stress (Table 3), indicating the destruction of the membrane stability and increasing the membrane lipid peroxidation [22]. The undesirable alterations in the membranes that underwent stress were identified as inorganic leakage, in which salt stress was demonstrated repeatedly to induce peroxidative damage to cellular plasma membranes [56]. However, pretreatment with antioxidants, especially sequenced AsA-Pro-GSH showed significant RWC and MSI improvements under NaCl-salt stress, preventing partially or the peroxidative damage to plasma membranes especially due to the fact that MSI value of cucumber tissue was reached equally to the MSI unstressed control value that was also true for the RWC value (Table 3). [57] showed that AsA treatment prevented the peroxidation of membrane lipids and reduced malondialdehyde production as a final product of membrane lipid peroxidation, positively modifying the membrane properties and functions to minimize inorganic leakage and consequently improve the stability of cell membranes [28]. The same results were explained both with Pro [58] and GSH [21]. RWC is a proper measurement of tissue status of water in the term physiological result of cellular water scarcity, while water potential is a measurement of plant water transport in the soil-plant-atmosphere continuum [59]. It is a key marker for various studies of salinity stress. It is also a general measurement used to assess the balance of water in plant tissues over periods of water deficit and measures the amount of leafy water in a plant as a portion of the total volumetric water, which can be held in the leaf at its full aqueous capacity. In plant cells, RWC maintaining allows the metabolic activities to continue by osmotic adjustments and other attributes of salinity and/or drought adaptation [60]. The use of AsA-Pro-GSH resulted in a significant increase of RWC, helping positive modifying of the plasma membrane that is reported as a target of unacceptable environmental stressors. As found with AsA-Pro-GSH treatment, it is often proved that maintaining cell membrane stability and integrity is a key component to achieving satisfactory plant performance [28,61]. The favorable effects of the sequential AsA-Pro-GSH treatment on MSI and RWC can be explained based on the integrative positive modification of osmotic adjustment by Pro addition and improving the efficiency of AsA-GSH cycle by AsA and GSH addition for scavenging the ROS effectively. Additionally, the advantageous effects, in this regard, of AsA, Pro, and GSH, which form a pivotal component of the abiotic stress response in plant cells [21,60].

Conferring several mechanisms to overcome salt stress effects, Pro, AsA, and GSH, as low-molecular-weight antioxidants with others, comprise a major part of the plant defense system, rendering safeguarding roles to withstand oxidative stress effects [62]. Due to the fact that Pro acts as osmo- and/or enzyme protectant and might make itself as a reserve of N, as well as it considers as a free radical scavenger, there is a potent connection between its level in plant cells and the ability to withstand the effects of stress. [63]. Our findings showed a noticeable proline level in cucumber transplants (Table 3), positively reflecting in transplant growth, tissue water content, and photosynthetic efficiency (Table 1 and Table 2) by AsA, Pro, and GSH pretreatment, especially when applied in a sequencing method. This result can be obtained on account of the expression of key genes-encoding enzymes for Pro biosynthesis (the biosynthetic P5CS) and oxidizing enzyme low activities [28,64]. In addition, Pro acts to modify toxic ions (Cd^2+^; Figure 1 and Na^+^; Figure 2) and organic solute contents [64]. It was also effective in districting the oxidative damages of NaCl by the plant’s antioxidant system, which includes various enzymes and low-molecular-weight antioxidants (Table 3 and Table 4). Another mechanism that may be acted in transplants under NaCl stress, the Pro/P5C cycle transfers the NAD(P)H equivalents, which are reduced to the mitochondria to support the maintenance of the pool of NAD(P)^+^ [65]. In the salt-stressed leaves of the plant, there is marked ProDH activity and decreased levels of Pro and P5C. These compounds (Pro and P5C) can act as sources of N due to the increase in soluble protein and N compound contents that contribute to the osmotic adjustment [64].

As shown in Table 3, AsA and GSH levels in AsA-, Pro-, and/or GSH-pretreated salt-stressed cucumber transplants were found to be markedly greater in comparison with the control levels. The AsA-Pro-GSH as an integrative treatment was more effective than individuals. These results are in a parallel line with those of [28]. Minutely, the pool of AsA and GSH must be balanced along with appropriate APX activity to improve the plant’s antioxidant ability to avoid damage of oxidative stress [66]. AsA is a highly potent ROS scavenger due to its electron donation ability in various reactions that occurred enzymatically and non-enzymatically. It keeps safe the cell membrane integrity by direct ROS (O_2_^•−^ and OH^−^) scavenging [67]. Since the AsA-GSH cycle contains both AsA and GSH as major components, they can control the level of H_2_O_2_ in plant cells. By forming AsA and GSH, GR together with MDHAR and DHAR the all are mostly responsible for providing substrates for APX [68]. Under the stress of salinity, Desoky et al. [69] have reported increases in the state of redox activity of AsA and GSH in conjunction with an increase in their contents to reduce the level of H_2_O_2_. The reactions occurring in the transformation of oxidized glutathione; catalyzes the reduction of oxidized glutathione to reduced glutathione; GSH are catalyzed by GR.

In addition to non-enzyme-based antioxidants that play important roles in counteracting the effects of salinity, plants make use of antioxidant enzymes. This has become evident that comparatively raised activities of enzymes that scavenge different ROS have been reached in wheat seedlings [69]. Results of the current study (Table 4) have supported this finding. Also, the antioxidative enzymes assayed in the present work such as CAT, APX, GR, and SOD have a special role in mitigating the effects of oxidative stress stimulated by NaCl salinity. The activities of these enzymes were measured in cucumber transplants under normal or 100 mM NaCl stress in response to AsA, Pro, or GSH (singly) or AsA-Pro-GSH (sequential method as antioxidative integration). Our results showed that the activities of all enzymes raised under the stress of NaCl salinity and further increased with antioxidants pretreatment, especially with AsA-Pro-GSH pretreatment. Because SOD is an effective O_2_^•–^ scavenger, it is the plant’s first defense employee against ROS [28], demonstrating the SOD defensive role for biosystems. Besides, CAT is considered to be the main scavenger of H_2_O_2_, producing H_2_O and O_2_. It may also be a protective agent against the formation of OH^–^ radicals, which peroxidize cell membrane lipids and severely affect plant growth [70]. As CAT does, APX eliminates H_2_O_2_, and its elevated activity has also been noticed under salinity in different plant species [25,69]. Salt stress stimulates excess ROS accumulation in plant cells such as H_2_O_2_, which its metabolism depends on several antioxidant enzymes that functionally interconnected to eliminate H_2_O_2_ from cells of stressed plants [71,72].

AsA, Pro, and GSH contribute to reduce the salt-induced K^+^ efflux and increase Ca^2+^/Na^+^ and K^+^/Na^+^ ratios in the transplants stressed with NaCl salinity (Figure 1 and Figure 2), conferring proper ionic homeostasis in salt-stressed transplants. This suggests an effective mechanism in the roots of transplants stressed with NaCl salinity to avert Na^+^ xylem tonnage. Another efficient mechanism that may occur by applied antioxidants is the compartmentalization of excess Na^+^ authorizing a maximal K^+^ influx to transplant leaves [24,73,74], leading to an increased cytosolic ratio of K^+^/Na^+^ (along with Ca^2+^/Na^+^ ratio) that represents a crucial indicator for tolerance to salinity stress in plants [69]. An adaptive mechanism such as elevated K^+^ re-uptake lets plant cell to avert starvation of K^+^ under increased salts. Also, the three applied antioxidants may have integrated crucial roles as the main mechanism in reducing Cd^2+^ content in transplants (Figure 1). This may be due to the partitioning of Cd^2+^ to different organs to overcome the toxicity impacts of Cd^2+^ in the transplants of cucumber. Exogenous AsA-Pro-GSH improved ROS removal and metal ions chelation activities, establishing an important portion of the plant cell response to abiotic stress [24].

In general, sequential pretreatment with AsA-Pro-GSH resulted in the best findings as compared to their individuals, attenuating the stress harmful impacts of NaCl salinity. Exogenously applied AsA-Pro-GSH as a sequential pretreatment has been proved to attenuate the stress adverse impacts of 100 mM NaCl salinity on metabolic processes related to plant growth [28]. These antioxidants also reduced the Na^+^ and Cd^2+^ ion contents within plants (Figure 1 and Figure 2) due to the decreased uptake of these injurious ions and/or compartmentalization of them into transplant organs.

If a look is taken at the potential plant salt tolerance mechanisms proved in several works and the supporting role of exogenous AsA-Pro-GSH pretreatment performed in the current study, it has been found that: (1) Accumulation of osmotic adjustment substances is an important salt tolerance mechanism. This mechanism is supported by the exogenous addition of Pro in the AsA-Pro-GSH application, wherein plant cells Pro is considered to be a primary substance for osmotic adjustment and is also acted as a scavenger of ROS, a buffer of redox reactions, and/or molecular chaperone. These potential functions of Pro contribute to stabilizing the structures of plasma membranes and proteins under the conditions of stress, reflecting positive results in our study. (2) Selective absorption of ions and their compartmentalization is another pivotal mechanism of tolerance to salinity stress in plants. A high cytosolic K+/Na+ ratio contributes to the cells emptying of Na+ ions or conveying them to the region of inactive metabolism, the translocation of Na^+^ ions to the extracellular zone by the Na^+^/H^+^ antiporter at plasma membranes, and/or the partitioning of Na^+^ by the Na^+^/H^+^ antiporter in the vacuoles [75]. Besides, the gene *A. thaliana* AtNHX1 encodes the Na1yH1 antiporter at the tonoplast and is functioned by compartmentalization of Na^+^ into the cell vacuoles [76]. These results are in a parallel line with our results concerning the reduction of Na^+^ ion and the increase of K^+^ ion and K^+^/Na^+^ ratio by AsA-Pro-GSH application. (3) Enzymatically or non-enzymatically scavenging of the ROS by various antioxidants is a very crucial salt tolerance mechanism. The exogenous application of AsA, Pro, and GSH (non-enzymatic, low-molecular-weight antioxidants) in the sequential AsA-Pro-GSH treatment significantly supported their endogenous concentrations to effectively scavenge the ROS, reflecting in positive results in our study. These important mechanisms may be supported by another crucial salt tolerance mechanism; (4) Salt tolerance genes. Tolerance to the effects of salinity stress is a polygenic genetic trait. The plasma membrane Na^+^/H^+^ antiporter gene SOS1 and vacuolar Na^+^/H^+^ antiporter gene AtNHX1 in *A. thaliana*, in addition to rice OsbZIP71 gene and wheat genes Ta-UPnP, TaZNF, TaSST, TaDUF1, and TaSP are proved to promote the tolerance to the impacts of salinity stress in transgenic plants [75,77,78].

Improvements occurred by exogenous antioxidants in the seedling photosynthetic efficiency (Table 2 and Table 5), the relative content of water and stability index of cell membranes (Table 3 and Table 5), endogenous levels of AsA, free proline, and glutathione (Table 3 and Table 5), and the activities of antioxidative enzymes (Table 4 and Table 5) positively affected the growth traits of salt-stressed cucumber seedlings. Besides, the increased K^+^ and Ca^2+^ contents contributed to higher ratios of Ca^2+^/Na^+^ and K^+^/Na^+^, which were associated with a markedly lower Na^+^ content with the exogenous use of AsA-Pro-GSH (Table 5; Figure 1 and Figure 2) and all finally contributed to the increases in growth traits of salt-stressed cucumber seedlings (Table 1 and Table 5).

## 5. Conclusions

When applied exogenously as individuals or sequentially to soaking the seeds of cucumber, AsA, Pro, and GSH significantly enhanced the transplant water content and the cell membranes stabilities. Besides, photosynthetic activity, nutrients contents, and antioxidant defense systems were also improved along with the decrease of Na^+^ and Cd^2+^ contents under 100 mM NaCl-salt stress. These positive findings have led to the healthy growth of cucumber transplants. Pretreatment with AsA-Pro-GSH applied sequentially was more effective than any of the three individuals; Pretreatment with AsA, Pro, or GSH. Therefore, the elevation of the tolerance to salinity stress impacts in the transplants of cucumber, and the improvement of transplant growth and health would occur with pretreatment with AsA-Pro-GSH applied sequentially upon growth under 100 mM NaCl-salt stress.

## Figures and Tables

**Table 1:** Effects of seed single or sequential application of antioxidants on growth traits of 100 mM NaCl-salt-stressed cucumber transplants.

Treatments	Shoot Length (cm)	Leaf Area (cm^2^)	Stem Diameter (mm)	Shoot FW (g)	Shoot DW (g)
Control	15.6 ± 0.4 ^cd^	64.0 ± 2.2 ^cd^	3.6 ± 0.1 ^b^	1.71 ± 0.07 ^b^	0.13 ± 0.02 ^d^
AsA	17.0 ± 0.6 ^abc^	70.6 ± 5.8 ^ab^	3.8 ± 0.1 ^ab^	2.00 ± 0.16 ^a^	0.15 ± 0.03 ^bc^
Pro	16.6 ± 0.7 ^bcd^	69.3 ± 3.6 ^bc^	3.7 ± 0.1 ^b^	2.03 ± 0.08 ^a^	0.15 ± 0.04 ^bc^
GSH	17.9 ± 0.7 ^ab^	70.8 ± 3.3 ^ab^	3.8 ± 0.1 ^ab^	2.02 ± 0.08 ^a^	0.15 ± 0.02 ^bc^
AsA-Pro-GSH	18.3 ± 0.6 ^a^	75.5 ± 5.0 ^a^	4.0 ± 0.1 ^a^	2.03 ± 0.14 ^a^	0.17 ± 0.04 ^a^
NaCl	11.8 ± 0.6 ^e^	47.2 ± 2.0 ^e^	3.0 ± 0.1 ^c^	1.41 ± 0.05 ^c^	0.10 ± 0.02 ^e^
NaCl + AsA	16.8 ± 0.3 ^abc^	63.4 ± 4.2 ^d^	3.6 ± 0.1 ^b^	1.95 ± 0.12 ^a^	0.14 ± 0.02 ^cd^
NaCl + Pro	15.4 ± 0.6 ^cd^	61.1 ± 2.3 ^d^	3.6 ± 0.1 ^b^	1.98 ± 0.06 ^a^	0.13 ± 0.02 ^d^
NaCl + GSH	15.1 ± 0.5 ^d^	64.1 ± 3.6 ^cd^	3.6 ± 0.1 ^b^	1.98 ± 0.09 ^a^	0.14 ± 0.03 ^cd^
NaCl + AsA-Pro-GSH	17.9 ± 0.4 ^ab^	70.7 ± 2.5 ^ab^	3.7 ± 0.1 ^b^	2.00 ± 0.07 ^a^	0.16 ± 0.04 ^ab^

Means with the same letter within each trait are not significantly differed at *p* ≤ 0.05. AsA = ascorbic acid, Pro = proline, GSH = glutathione, and AsA-Pro-GSH = sequential application of different antioxidants.

**Table 2:** Effects of seed single or sequential application of antioxidants on photosynthetic efficiency of 100 mM NaCl-salt-stressed cucumber transplants.

Treatments	Stomatal Conductance (mmol^−2^ S^−1^)	SPAD Chlorophyll	F_v_/F_m_	Performance Index (%)
Control	193 ± 5 ^d^	42.1 ± 2.2 ^ab^	0.83 ± 0.06 ^a^	3.64 ± 0.24 ^ab^
AsA	241 ± 7 ^abc^	42.4 ± 1.0 ^ab^	0.83 ± 0.05 ^a^	3.75 ± 0.10 ^ab^
Pro	247 ± 5 ^abc^	42.8 ± 0.6 ^ab^	0.83 ± 0.05 ^a^	3.73 ± 0.12 ^ab^
GSH	254 ± 6 ^a^	42.9 ± 0.9 ^ab^	0.83 ± 0.04 ^a^	3.78 ± 0.14 ^a^
AsA-Pro-GSH	257 ± 8 ^a^	44.7 ± 1.4 ^a^	0.84 ± 0.05 ^a^	3.78 ± 0.12 ^a^
NaCl	142 ± 4 ^e^	33.8 ± 2.0 ^e^	0.76 ± 0.04 ^b^	3.03 ± 0.35 ^c^
NaCl + AsA	232 ± 8 ^c^	38.9 ± 1.8 ^cd^	0.82 ± 0.05 ^a^	3.46 ± 0.30 ^b^
NaCl + Pro	234 ± 5 ^bc^	38.6 ± 1.9 ^d^	0.82 ± 0.04 ^a^	3.46 ± 0.29 ^b^
NaCl + GSH	234 ± 7 ^bc^	39.0 ± 0.8 ^cd^	0.82 ± 0.04 ^a^	3.48 ± 0.31 ^b^
NaCl + AsA-Pro-GSH	252 ± 6 ^ab^	41.4 ± 1.5 ^bc^	0.84 ± 0.05 ^a^	3.72 ± 0.19 ^ab^

Means with the same letter within each trait are not significantly differed at *p* ≤ 0.05. AsA = ascorbic acid, Pro = proline, GSH = glutathione, and AsA-Pro-GSH = sequential application of different antioxidants. F_v_/F_m_ = maximum quantum yield of PSII = efficiency of the photosystem 2.

**Table 3:** Effects of seed single or sequential application of antioxidants on leaf relative water content (RWC), membrane stability index (MSI), and non-enzymatic antioxidant system of cucumber transplants grown under 100 mM NaCl-salt stress.

Treatments	RWC (%)	MSI (%)	Free Proline (mg g^−1^ DW)	AsA (μmol Ascorbate g^−1^ DW)	GSH (nmol GSH g^-1^ DW)
Control	87.6 ± 0.7 ^abc^	76.3 ± 1.1 ^a^	0.27 ± 0.00 ^f^	1.48 ± 0.04 ^g^	0.14 ± 0.01 ^g^
AsA	88.3 ± 1.0 ^abc^	78.5 ± 0.4 ^a^	0.31 ± 0.01 ^ef^	3.54 ± 0.08 ^d^	0.19 ± 0.01 ^f^
Pro	89.2 ± 0.8 ^a^	78.3 ± 0.5 ^a^	0.65 ± 0.04 ^c^	1.85 ± 0.02 ^f^	0.20 ± 0.00 ^f^
GSH	88.7 ± 2.1 ^ab^	79.0 ± 1.1 ^a^	0.32 ± 0.02 ^e^	1.97 ± 0.04 ^f^	0.35 ± 0.01 ^d^
AsA-Pro-GSH	91.3 ± 2.6 ^a^	79.7 ± 0.8 ^a^	0.76 ± 0.00 ^b^	4.27 ± 0.08 ^c^	0.40 ± 0.00 ^c^
NaCl	74.5 ± 0.4 ^d^	63.6 ± 2.1 ^c^	0.33 ± 0.00 ^e^	2.89 ± 0.08 ^e^	0.18 ± 0.01 ^fg^
NaCl + AsA	82.7 ± 0.9 ^c^	69.8 ± 0.7 ^b^	0.40 ± 0.02 ^d^	6.32 ± 0.05 ^b^	0.25 ± 0.00 ^e^
NaCl + Pro	83.2 ± 2.7 ^bc^	69.9 ± 1.5 ^b^	0.80 ± 0.01 ^b^	3.34 ± 0.03 ^d^	0.28 ± 0.01 ^e^
NaCl + GSH	82.8 ± 1.2 ^bc^	70.4 ± 0.8 ^b^	0.41 ± 0.01 ^d^	3.40 ± 0.04 ^d^	0.49 ± 0.01 ^b^
NaCl + AsA-Pro-GSH	87.8 ± 1.4 ^abc^	76.8 ± 1.0 ^a^	0.87 ± 0.01 ^a^	7.58 ± 0.13 ^a^	0.59 ± 0.01 ^a^

Means with the same letter within each trait are not significantly differed at *p* ≤ 0.05. AsA = ascorbic acid, Pro = proline, GSH = glutathione, and AsA-Pro-GSH = sequential application of different antioxidants.

**Table 4:** Effects of seed single or sequential application of antioxidants on enzymatic antioxidant system of cucumber transplants grown under 100 mM NaCl-salt stress.

Treatments	Superoxide Dismutase	Catalase	Glutathione Reductase	Ascorbate Peroxidase
	μmol mg^‒1^ Protein min^‒1^			
Control	0.23 ± 0.01 ^e^	0.23 ± 0.00 ^d^	0.27 ± 0.01 ^d^	0.30 ± 0.01 ^e^
AsA	0.29 ± 0.01 ^cd^	0.28 ± 0.01 ^c^	0.32 ± 0.01 ^c^	0.43 ± 0.02 ^c^
Pro	0.28 ± 0.01 ^d^	0.30 ± 0.02 ^c^	0.34 ± 0.02 ^c^	0.42 ± 0.01 ^c^
GSH	0.28 ± 0.01 ^d^	0.28 ± 0.01 ^c^	0.34 ± 0.02 ^c^	0.42 ± 0.02 ^c^
AsA-Pro-GSH	0.31 ± 0.02 ^bcd^	0.35 ± 0.00 ^b^	0.41 ± 0.02 ^b^	0.52 ± 0.02 ^b^
NaCl	0.25 ± 0.01 ^e^	0.29 ± 0.00 ^c^	0.32 ± 0.01 ^c^	0.36 ± 0.01 ^d^
NaCl + AsA	0.31 ± 0.01 ^bcd^	0.35 ± 0.01 ^b^	0.39 ± 0.02 ^b^	0.49 ± 0.02 ^b^
NaCl + Pro	0.31 ± 0.02 ^bcd^	0.37 ± 0.00 ^b^	0.39 ± 0.01 ^b^	0.50 ± 0.02 ^b^
NaCl + GSH	0.33 ± 0.02 ^b^	0.37 ± 0.01 ^b^	0.40 ± 0.02 ^b^	0.50 ± 0.02 ^b^
NaCl + AsA-Pro-GSH	0.38 ± 0.02 ^a^	0.41 ± 0.02 ^a^	0.47 ± 0.02 ^a^	0.55 ± 0.02 ^a^

Means with the same letter within each trait are not significantly differed at *p* ≤ 0.05. AsA = ascorbic acid, Pro = proline, GSH = glutathione, and AsA-Pro-GSH = sequential application of different antioxidants.

**Table 5:** Changes (%) in seedling physiological, biochemical and growth traits, and antioxidant system components relative to the control in cucumber under normal (N) and saline (100 mM NaCl) conditions. Three color scale heatmap as follow: yellow = midpoint of control and different traits with insignificant values compared to control, green = changes over control, and red = changes below control.

Parameters	Control (N)	Treatments								
		N+ AsA	N+ Pro	N+ GSH	N+ A.P.G	NaCl (S)	S+ AsA	S+ Pro	S+ GSH	S+ A.P.G
Shoot length	15.6	9.0	6.4	15	17	−24	7.7	−1.3	−3.2	15
Leaf area	64.0	10	8.3	11	18	−26	−0.9	−4.5	0.2	10
Stem diameter	3.6	5.6	2.8	5.6	11	−17	0.0	0.0	0.0	2.8
Shoot FW	1.71	17	19	18	19	−18	14	16	16	19
Shoot DW	0.13	15	15	15	31	−23	7.7	0.0	7.7	23
gs	193	25	28	32	33	−26	20	21	21	31
SPAD value	42.1	0.7	1.7	1.9	6.2	−20	−7.6	−8.3	-7.4	-1.7
Fv/Fm	0.83	0.0	0.0	0.0	1.2	−8.4	−1.2	−1.2	−1.2	1.2
PI	3.64	3.0	2.5	3.8	3.8	−17	−4.9	-4.9	−4.4	2.2
RWC	87.6	0.8	1.8	1.3	4.2	−15	−5.6	−5.0	−5.5	0.2
MSI	76.3	2.9	2.6	3.5	4.5	−17	−8.5	−8.4	−7.7	0.7
Free proline	0.27	15	141	19	181	22	48	196	52	222
Ascorbate	1.48	139	25	33	189	95	327	126	130	412
Glutathione	0.14	36	43	150	186	29	79	100	250	321
SOD activity	0.23	26	22	22	35	8.7	35	35	43	65
CAT activity	0.23	22	30	22	52	26	52	61	61	78
GR activity	0.27	19	26	26	52	19	44	44	48	74
APX activity	0.30	43	40	40	73	20	63	67	67	83
Cd^2+^ content	0.53	−31	−2.5	−5.6	-33	550	283	292	257	94
K^+^ content	23.8	2.3	1.0	2.4	3.4	−55	−28	−28	−22	−1.9
Ca^2+^ content	13.5	−0.4	-2.5	0.9	1.2	−31	-16	−18	−13	−2.8
Na^+^ content	2.73	−2.8	−1.1	−1.9	-2.5	529	253	248	245	129
K^+^/Na^+^ ratio	8.67	5.4	2.2	4.4	6.2	−93	−80	−79	−77	−57
Ca^2+^/Na^+^ ratio	4.90	2.7	−2.6	2.8	3.9	−89	−77	−76	−75	−56

AsA = ascorbate, Pro = proline, GSH = glutathione, A.P.G. = ascrobate-proline-glutathione, gs = stomatal conductance, PI = performance index, RWC = relative water content, MSI = membrane stability index, CAT = catalase, GR = glutathione reductase, SOD = superoxide dismutase, APX = ascorbate peroxidase, Cd^2+^ = cadmium, Ca^2+^ = calcium, K^+^ = potassium, and Na^+^ = sodium.
